# Cost effectiveness analysis of afatinib versus pemetrexed-cisplatin for first-line treatment of locally advanced or metastatic EGFR mutation positive non-small-cell lung cancer from the Singapore healthcare payer’s perspective

**DOI:** 10.1186/s12885-018-4223-y

**Published:** 2018-03-27

**Authors:** Ping-Tee Tan, Mohamed Ismail Abdul Aziz, Fiona Pearce, Wan-Teck Lim, David Bin-Chia Wu, Kwong Ng

**Affiliations:** 1 Agency for Care Effectiveness, Ministry of Health, Harrower Hall, 14 College Road, Singapore, 169853 Singapore; 2 Division of Medical Oncology, National Cancer Centre Singapore, 11 Hospital Drive, Singapore, 169610 Singapore

## Abstract

**Background:**

Non-small-cell lung cancer (NSCLC) accounts for 85% of all lung cancers and is associated with a poor prognosis. Afatinib is an irreversible ErbB family blocker recommended in clinical guidelines as a first-line treatment for NSCLC which harbours an epidermal growth factor receptor (EGFR) mutation. The objective of this study was to evaluate the cost-effectiveness of afatinib versus pemetrexed-cisplatin for first-line treatment of locally advanced or metastatic EGFR mutation positive NSCLC in Singapore.

**Methods:**

A partitioned survival model with three health states (progression-free, progressive disease and death) was developed from a healthcare payer perspective. Survival curves from the LUX-Lung 3 trial (afatinib versus pemetrexed-cisplatin chemotherapy) were extrapolated beyond the trial period to estimate the underlying progression-free survival and overall survival parametric distributions. Rates of adverse reactions were also estimated from LUX-Lung 3 while health utilities from overseas were derived from the literature in the absence of local estimates. Direct costs were sourced from public healthcare institutions in Singapore. Incremental cost-effectiveness ratios (ICERs) were calculated over a 5 year time horizon. Deterministic and probabilistic sensitivity analyses and additional scenario analyses were conducted to explore the impact of uncertainties and assumptions on the cost-effectiveness results.

**Results:**

In the base-case analysis, the ICER for afatinib versus pemetrexed-cisplatin was SG$137,648 per quality-adjusted life year (QALY) gained and SG$109,172 per life-year gained. One-way sensitivity analysis showed the ICER was most sensitive to variations in the utility values, the cost of afatinib and time horizon. Scenario analyses showed that even reducing the cost of afatinib by 50% led to a high ICER which was unlikely to represent a cost-effective use of healthcare resources.

**Conclusions:**

Compared with pemetrexed-cisplatin, afatinib is not cost-effective as a first-line treatment for advanced EGFR mutation-positive NSCLC in Singapore. The findings from our study will be useful to inform local healthcare decision-making and resource allocations for NSCLC treatments, together with other considerations such as clinical effectiveness, safety and affordability of TKIs.

## Background

Lung cancer is one of the most common cancers diagnosed in adults in Singapore, accounting for approximately 11% of all cancers and 22% of cancer-related deaths from 2011 to 2015 [1]. Approximately 85% of all lung cancers are classified as non-small-cell tumours and the majority of patients have advanced or metastatic disease (stage IIIB/IV) at diagnosis [2].

Owing to genetic advancement, mutations in the epidermal growth factor receptor (EGFR), which play a role in tumour development and progression, have been found in a subset of lung adenocarcinomas and have led to a paradigm shift in therapy. The incidence of EGFR mutation is 10–20% in Caucasian populations [3] but as high as 50% in Asian patients [4]. Tyrosine kinase inhibitors (TKIs), such as erlotinib, gefitinib and afatinib have been developed to selectively inhibit EGFR tyrosine kinase activity, and in turn, prevent tumour growth and increase tumour cell apoptosis [5, 6]. The European Society for Medical Oncology (ESMO) and the Singapore Cancer Network (SCAN) recommend EGFR mutation testing for all patients with advanced NSCLC of non-squamous subtype [3, 7]. Both guidelines also recommend TKIs for the first-line treatment of advanced NSCLC harbouring an EGFR mutation [3, 7]. Local clinical experts confirm that the recommendations in these guidelines constitute routine clinical practice in Singapore for patients with NSCLC.

To date, none of the TKIs have been shown to significantly improve overall survival (OS) when compared with standard chemotherapy. Randomised controlled trials however have shown TKIs significantly improved progression-free survival (PFS) over standard chemotherapy in treatment-naïve patients with advanced EGFR mutation positive NSCLC [5, 8–10]. Patients treated with afatinib have also been reported to have better progression-free survival (PFS) compared to patients treated with gefitinib, but the absolute difference was small [11].

In local clinical practice, pemetrexed-platinum chemotherapy is the preferred platinum doublet used as an alternative to TKIs in view of its better clinical outcomes compared with other chemotherapy regimens [7]. There is currently only one published randomised trial (LUX-Lung 3) that compares a TKI (afatinib) with pemetrexed-based chemotherapy for the first-line treatment of advanced EGFR mutation positive NSCLC [6].

For decision-makers, the choice between TKIs or chemotherapy is largely influenced by comparative effectiveness and costs. Therefore, the objective of this study was to evaluate the cost-effectiveness of afatinib versus pemetrexed-cisplatin for first-line treatment of locally advanced or metastatic EGFR mutation positive NSCLC to inform local drug subsidy decisions in Singapore.

## Methods

### LUX-lung 3 trial

LUX-Lung 3 was a global, randomised, open-label phase III trial comparing first-line afatinib (*n* = 229; 40 mg once daily) with pemetrexed plus cisplatin (*n* = 111; PemCis) chemotherapy (500 mg/m^2^ pemetrexed and 75 mg/m^2^ cisplatin once every 21 days for a maximum of 6 cycles) in patients with advanced lung adenocarcinoma with proven EGFR mutations. Treatment arms were balanced in terms of patient demographics and clinical characteristics. Approximately 65% of patients were women, 68% were never-smokers and 72% were East Asian ethnicity. The efficacy endpoints included progression-free survival (PFS) (defined as time from random assignment to disease progression or death), objective response rate (ORR) and overall survival (OS).

Median PFS (investigator-reviewed) for afatinib was 11.1 months compared with 6.7 months for PemCis (hazard ratio [HR] 0.49; 95% confidence interval [CI]: 0.37 to 0.65; *p* = 0.001) [6]. OS did not differ between afatinib and PemCis in the overall study population after median follow-up of 41 months (HR 0.88; 95% CI: 0.66 to 1.17; *p* = 0.39) [12]. Diarrhoea and rash were the most common treatment related adverse events (AEs) in patients receiving afatinib, while nausea, fatigue, decreased appetite and myelosuppression were most commonly associated with PemCis [6].

### Model structure and outcomes

#### Model structure

**Fig. 1:**
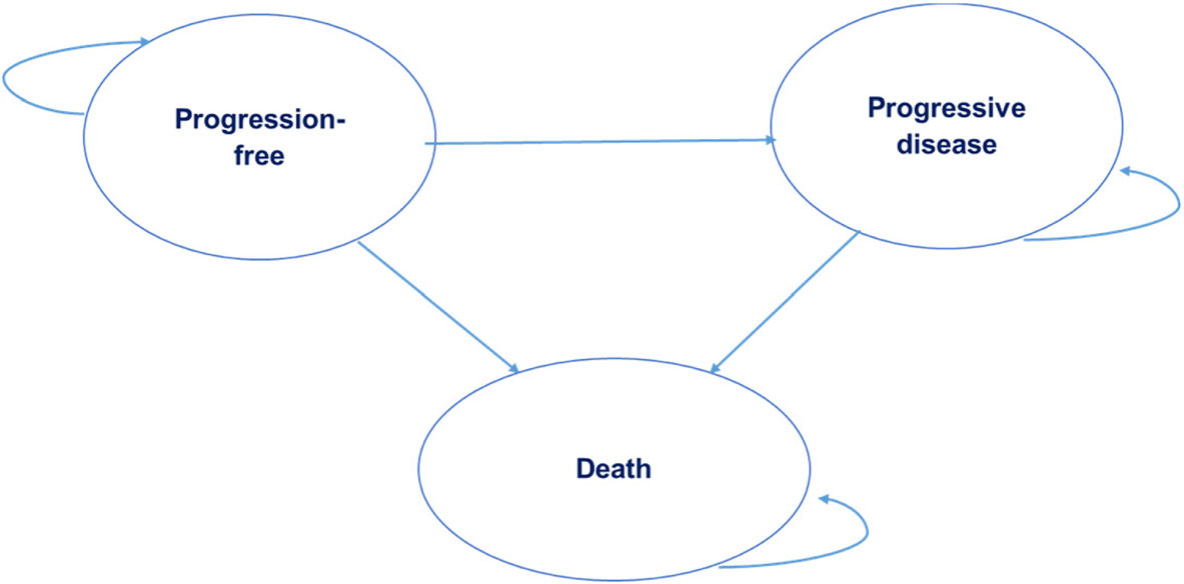
Partition survival model with three health states

An excel-based partitioned survival model (PSM) was developed to assess the cost-effectiveness of afatinib compared with pemetrexed-cisplatin (PemCis) chemotherapy for the first-line treatment of patients with locally advanced or metastatic EGFR mutation positive NSCLC. The model included three health states: progression-free (PF), progressive disease (PD) and death (Fig. 1). All patients were assumed to enter the model in the PF health state and could either remain in the same health state or transition to the PD or Death state at the beginning of each cycle [13]. Patients who progressed to the PD state could stay within the same health state or progress to death; but not revert back to the PF state. The model had a time horizon of 5 years in the base-case, and a cycle length of 1 month (including a half-cycle correction).

#### Treatment pathway

The PSM compared two different first-line treatment arms as reflected in the LUX-lung 3. For the intervention arm, patients were assumed to receive afatinib (first-line), then PemCis (second-line) when their disease progressed, before moving to best supportive care (BSC) upon further progression. For the comparator arm, patients received PemCis (first-line), then afatinib (second-line) when their disease progressed, followed by BSC. Dosing regimens were based on the recommended dosages in the package inserts for each treatment and in line with LUX-Lung 3. Dose of PemCis was calculated assuming body surface area (BSA) of 1.6m^2^. Local oncologists were consulted to ensure the treatment algorithm in the analysis reflected routine clinical practice for the management of NSCLC in Singapore.

#### Outcomes

Analyses were conducted from the Singapore healthcare payer’s perspective. The outcomes of interest were progression-free life years (PFLYs), overall life years (LYs), quality-adjusted life years (QALYs), the total cost of intervention and comparator treatments, as well as the incremental cost-effectiveness ratio (ICER). A discount rate of 3% was applied to both costs and outcomes.

### Model parameters

#### Clinical efficacy data

The population enrolled in LUX-Lung 3, i.e. treatment-naïve patients with stage IIIB and IV advanced EGFR mutation positive lung adenocarcinoma is reflective of the population of interest for subsidy consideration of TKIs in Singapore [6, 12]. The proportion of patients in each health state was derived from clinical trials [6, 12–14]. The area under curve (AUC) from LUX-Lung 3 was used to determine the mean time that patients remained in each health state.

OS and PFS for patients receiving first-line treatments were extrapolated from LUX-Lung 3 (Table 1). To do this, individual data points from the Kaplan-Meier (KM) curves for OS and PFS (investigator-reviewed) in the published paper were extracted using the WebPlotDigitizer developed by Rohatgi [15]. Then, a curve fitting approach developed by Hoyle and Henley [16] was used to estimate the underlying survival distribution from the digitised KM graphs. The curve fitting approach (with online Microsoft Excel spreadsheet and R statistics code) used survival probabilities to estimate the number of patients with events and the number censored in each time interval as a proxy for individual patient data (IPD). The tail-ends of the curves were fitted with various parametric survival models i.e. exponential, Weibull, log-logistic and log-normal. Weilbull model produced the best goodness of fit to the observed survival data based on the Akaike Information Criterion (AIC) value. Reduction factors were incorporated when combining the observed data and the extrapolated tail-end data [17].

**Table 1:** Clinical efficacy data from LUX-Lung 3

	Afatinib arm	Pemetrexed-cisplatin arm
Median overall survival, months (95% CI)	28.2 (24.6–33.6)^b^	28.2 (20.7–33.2)^b^
Median first-line progression free survival, months^a^	11.1^c^	6.7^c^

*CI* confidence interval

^a^95%CI was not reported

Source: ^b^ Yang et al., [12]; ^c^Sequist et al, [6]

In the PD health state, patients were assumed to cross over to second-line treatment. The time spent in PD was derived from the difference between AUCs of OS and first-line PFS. The proportion of time that patients received second-line treatment was derived from the literature. The median PFS was 11.9 months for afatinib (assumed from the PFS of combined gefitinib and erlotinib arms in Kim et al. [14]) and 5.4 months, for PemCis (from Soria et al [13]). The time spent receiving second-line treatment was assumed to remain constant across both arms irrespective of the relative time spent in the PD state.

During the remaining time in PD, patients could receive BSC as third-line therapy. The time was calculated, for each arm, by subtracting the time spent on second-line therapy from the estimated total time spent in PD (Table 2).

**Table 2:** Time spent receiving each treatment during PD health state

	Afatinib arm	Pemetrexed-cisplatin arm
Estimated total time in PD, months	21.17^a^	23.92^a^
Mean time receiving second-line treatment, months	5.40^c^	11.90^d^
Mean time receiving third-line treatment (BSC), months	15.77^b^	12.02^b^

*PD* progressive disease

^a^From partitioned survival model

^b^Time in BSC = time in PD – time in second-line

Source: ^c^ Soria et al, [13]; ^d^Kim et al, [14]

#### Adverse events

Treatment related AEs grade ≥ 3 in LUX-Lung 3 were incorporated in the model. Based on local expert opinion, only AEs that had a substantial impact on patients’ quality-of-life and cost of AE management were included (Table 3). The model assumed AEs occurred mutually exclusive of each other. The duration of each AE was estimated from expert opinion.

**Table 3:** Incidence of grade ≥ 3 adverse events

Grade ≥ 3 adverse events	Afatinib (%)	Pemetrexed-cisplatin (%)
Neutropenia	0.4	18.0
Fatigue	1.3	12.6
Anaemia	0.4	6.3
Nausea	0.9	3.6
Diarrhoea	14.4	0.0
Rash	16.2	0.0
Vomiting	3.1	2.7

Source: Sequist et al, [6]

#### Cost

Only direct costs were incorporated into the model including the cost of drugs, consultation visit, monitoring, BSC, and managing AEs (Table 4).

**Table 4:** Unit costs included in the model

	Cost (SG$)	Range (SG$)	Source
Cost of drugs^a^			
Afatinib (per 40 mg tablet)^b^	102.95	98.80 to 104.29	[f]
Pemetrexed (per 500 mg vial)^b^	440.54	327.80 to 562.30	[f]
Cisplatin (per 50 mg vial)^b^	15.61	12.45 to 18.50	[f]
Cost of chemotherapy administration			
Facility fee/chair time (2 to 4 h)^c^	272.20	241.00 to 319.00	[f]
Chemotherapy preparation fee by pharmacy	52.80	12.00 to 80.00	[f]
Cost of consultation visit and monitoring			
Consultation visit	74.57	93.00 to 102.72	[f]
Computerised tomography-thorax scan	940.00	850.00 to 1000.00	[f]
Liver function test	71.30	52.40 to 83.90	[f]
Full blood count test	26.46	24.00 to 28.10	[f]
Renal panel test	62.80	35.20 to 81.20	[f]
Cost of best supportive care (BSC)			
Inpatient hospice stay (per day)	275.00	–	[g]
Home hospice visit	Nil^d^	–	[g]
Cost of managing adverse event			
Anaemia (per episode)^e^	1486.00	–	[h]
Diarrhoea (per episode)^e^	1382.40	–	[h]

^a^Cost of drug is based on the selling price to patient

^b^Dosing regimens were based on recommended dosages in package inserts for afatinib (40 mg/day), pemetrexed (500 mg/m^2^/cycle) and cisplatin (75 mg/m^2^/cycle) and assumed no vial sharing. Up to 6 cycles of chemotherapy, every 21 days, were allowed. An average Body Surface Area (BSA) of 1.6m^2^ was assumed

^c^Chair time for chemotherapy is approximately 2 h and 40 min. Pemetrexed is infused over 10 min, followed by 30 min break before infusion of cisplatin over 2 h approximately

^d^Home hospice visit is complementary from the hospice centre

^e^Included hospital admission charges and treatment cost

Source: ^f^ weighted average selling price across public healthcare institutions in Singapore; ^g^ price charged by one hospice centre in Singapore; ^h^ inpatient bill sizes [18, 19]

The cost of afatinib and PemCis chemotherapy was estimated from the weighted average selling price across public healthcare institution in Singapore. For each PemCis chemotherapy cycle, facility fee (chemotherapy chair time of 2 to 4 h) and chemotherapy preparation fee by the pharmacy were added to the drug cost. No vial sharing for PemCis was allowed in the analysis.

Advice on frequency and types of relevant outpatient consultation visits, monitoring scans and laboratory tests for patients were sought from local oncologists. Costs for consultation visit, computerised tomography-thorax (CT) scan, liver function test, full blood count and renal panel test were sourced from public healthcare institutions.

It was assumed that patients could receive BSC at home or in hospice centres. The distribution of patients across each setting (58.3% in home care; 41.7% in hospice centre) was estimated from expert opinion.

Cost of grade ≥ 3 AEs were included only if the AEs necessitated inpatient hospitalisation (i.e. anaemia and diarrhoea) because it was assumed inpatient costs would be greater. The costs of AEs were sourced from inpatient bill sizes (including hospital admission charges and treatment costs) from public hospitals [18, 19]. The duration to resolve each AE was estimated by expert opinion.

#### Utility values

In the absence of local data, utility values for the health states for each treatment arm were extracted from a prospective, international, quality-of-life survey of patients with advanced NSCLC receiving first-, second-, or third−/fourth-line pharmacotherapy or BSC [20]. Utilities were weighted by the proportion of time spent in the health states, and disutility of AEs (obtained from the UK general population [21]) was also applied (Table 5).

**Table 5:** Utility values for the health states and disutility values associated with adverse events

	Utility value	Range	Distribution	Source
Progression-free (no AE)				
1st -line afatinib and pemetrexed-cisplatin	0.71	0.67 to 0.76	Beta	[c]^a^
Progressive disease (no AE)				
2nd -line afatinib and pemetrexed-cisplatin	0.67	0.59 to 0.75	Beta	[c]^a^
3rd-line/ Best supportive care	0.59	0.42 to 0.77	Beta	[c]^a^
Adverse events				
Neutropenia	−0.090	0.059 to 0.120	Beta	[d]
Fatigue	−0.074	0.037 to 0.110	Beta	[d]
Anaemia^b^	−0.074	0.037 to 0.110	Beta	[d]
Nausea & vomiting	−0.048	0.016 to 0.080	Beta	[d]
Diarrhoea	−0.047	0.016 to 0.077	Beta	[d]
Rash	−0.033	0.010 to 0.055	Beta	[d]

^a^The utility was then weighted by the proportion of time spent in the health state

^b^Assumed from the disutility for fatigue

Source: ^c^Chouaid et al, [20]; ^d^Nafees et al, [21]

### Sensitivity analyses

One-way sensitivity analyses were conducted to explore the impact of uncertain model parameters on the ICER. Each parameter was varied independently by the lower and upper range of the 95% confidence interval or the range reported in literature.

A probabilistic sensitivity analysis (PSA) was also performed to further explore the uncertainty of input parameters by random sampling the parameters from assigned distributions. Probability distributions were selected in accordance to the nature of the variable. PFS and OS were sampled from multivariate normal distributions using the Cholesky decomposition matrix of the Weilbull distribution, whereas utility values were assumed to have a beta distribution (continuous distribution confined within interval 0 and 1). Monte Carlo simulations were repeated over 10,000 iterations to generate a distribution of ICER outcome shown as a scatterplot. There is no fixed willingness to pay (WTP) threshold to determine cost-effectiveness in Singapore, therefore a cost-effectiveness acceptability curve (CEAC) was generated to show the probability of afatinib being cost-effective across a range of WTP thresholds.

### Additional scenario analyses

Additional analyses were conducted to test the impact of different survival curve extrapolation approaches and pricing scenarios on the base case ICER. Instead of fitting the tail-end of the extrapolation KM survival curves with a Weilbull model (base case), the entire survival curves were fitted with different parametric distributions. Price discounts ranging from 10 to 50% for afatinib were also tested to simulate the potential cost savings to patients through a manufacturer’s patient assistance program in Singapore.

## Results

### Base-case analysis

In the base-case with a time horizon of 5 years, the ICER for afatinib versus PemCis was SG$137,648 per QALY gained and SG$109,172 per LY gained (Table 6). The afatinib arm led to more QALYs gained compared to the PemCis arm (1.69 versus 1.58 QALYs, respectively) at an incremental cost of SG$15,227.

**Table 6:** Summary of costs and benefits of afatinib vs PemCis - base-case analysis

	Afatinib	Pemetrexed-cisplatin	Incremental difference
Total cost (SG$)	93,958	78,731	15,227
Cost of PF state	44,205	11,236	32,969
Cost of PD state	49,548	67,401	−17,853
Cost of AE	205	94	111
Total benefit			
QALYs	1.69	1.58	0.11
LYs	2.59	2.45	0.14
PFLYs	1.18	0.68	0.50
ICER (QALY)	–	–	137,648
ICER (LY)	–	–	109,172

*ICER* incremental cost-effectiveness ratio, *QALY* quality-adjusted life year, *LY* life year, *PFLY* progression-free life year, *AE* adverse event, *PF* progression-free, *PD* progressed disease

### Sensitivity analyses

**Fig. 2:**
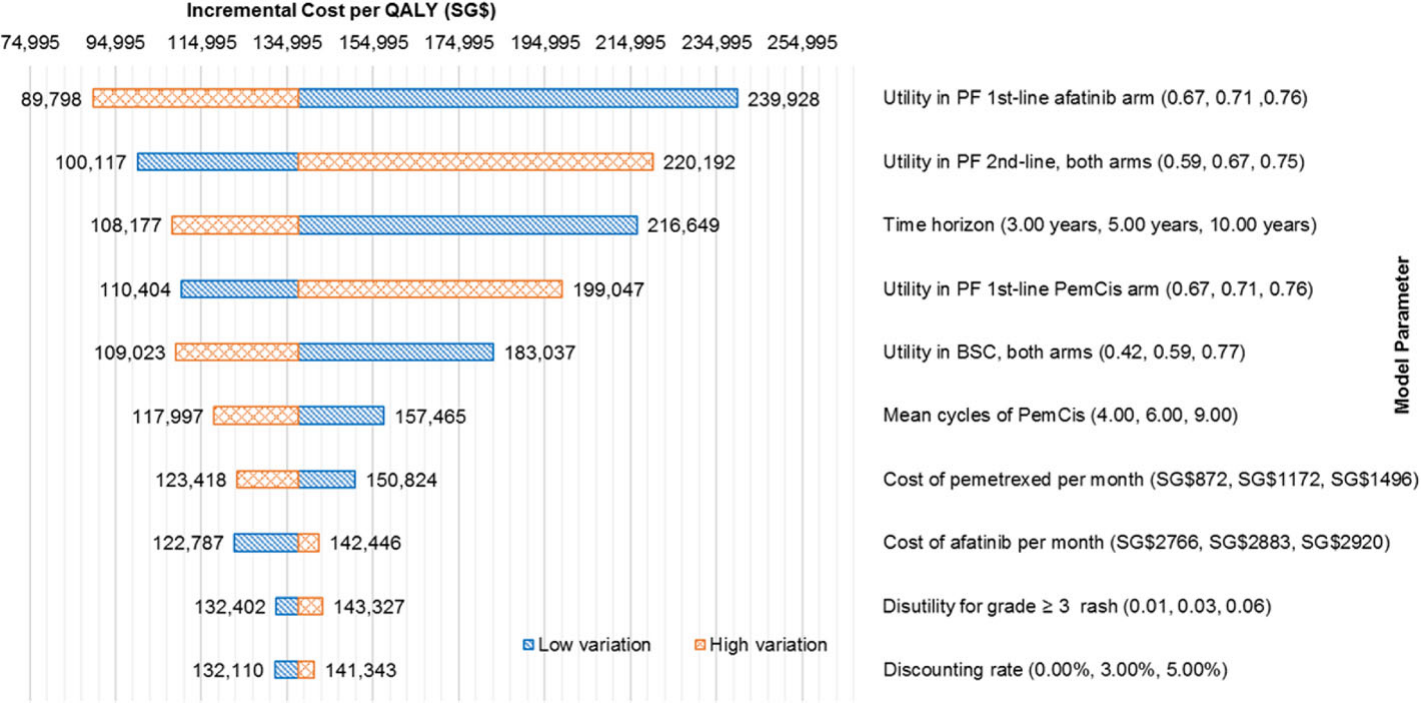
One way sensitivity analysis tornado diagram. QALY: quality-adjusted life year; PF: progression-free; PD: progressed disease; BSC: best supportive care; PemCis: pemetrexed-cisplatin

One-way sensitivity analyses confirmed that the ICER was most sensitive to variations in the utility values of PF assumed for the first-line and second-line treatments, and the time horizon of the model (Fig. 2). Using the lower range of utility value for the PF state for first-line afatinib increased the ICER to SG$239,928 per QALY, while applying the upper range of the utility value reported in the literature reduced the ICER to SG$89,798 per QALY. Shortening the time horizon to 3 years substantially increased the ICER to SG$217,175 per QALY, whereas lengthening the time horizon to 10 years reduced the ICER to SG$100,632 per QALY. Varying the discount rate and disutility associated with AEs had less impact on the ICER result.

**Fig. 3:**
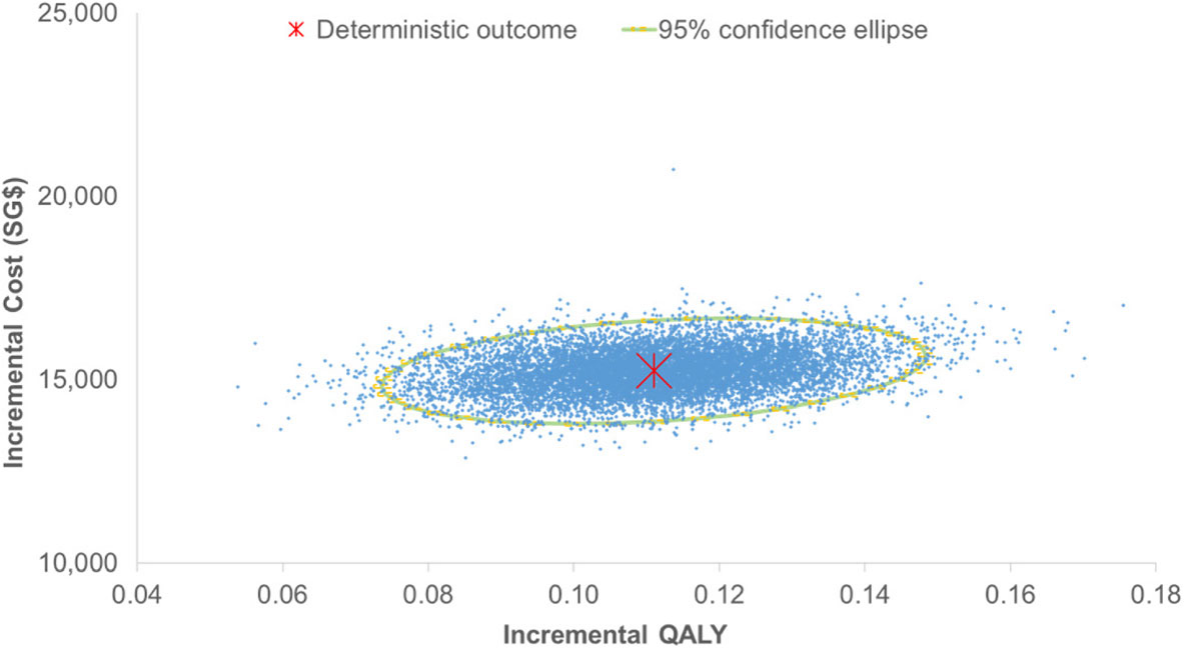
Probabilistic cost-effectiveness scatterplot. Each dot represents the ICER for 1 simulation

**Fig. 4:**
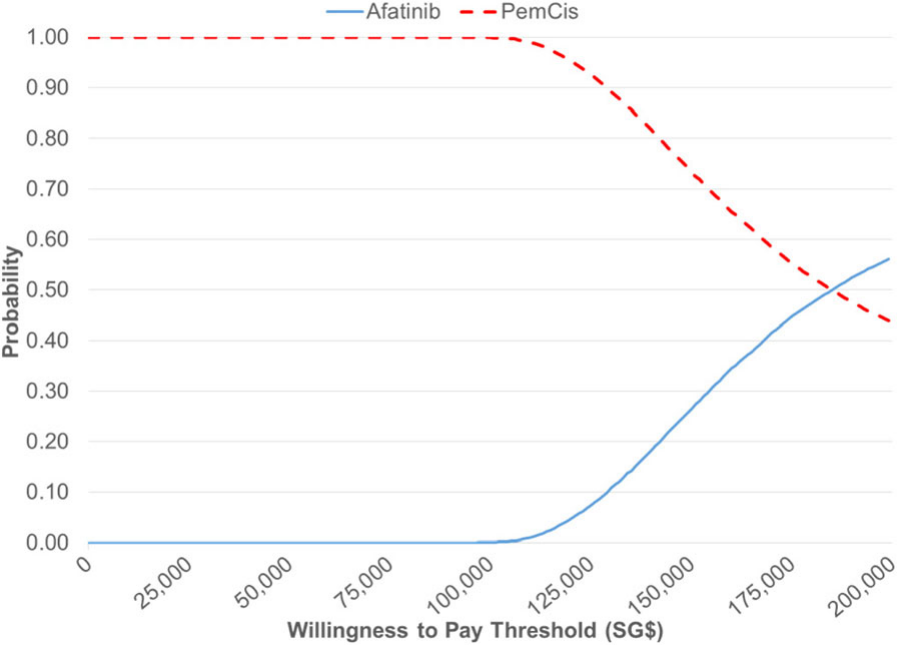
CEAC showing the likelihood of afatinib being cost-effective compared to pemetrexed-cisplatin across different WTP thresholds. CEAC: cost-effectiveness acceptability curve; WTP: willingness to pay

The PSA result was congruent with the base-case analysis where afatinib was consistently more effective and also more costly than PemCis in all 10,000 simulations (Fig. 3). The probabilistic ICER for afatinib versus PemCis was SG$137,391 per QALY. The CEAC (Fig. 4) showed that afatinib had zero probability of being cost-effective when the WTP threshold was below SG$110,000 per QALY and that there was only a 50% likelihood of it being more cost-effective than PemCis at a WTP threshold of SG$186,000 per QALY.

### Additional analyses

Results for the additional scenario analyses are shown in Table 7. Applying the Weilbull parametric fit to the entire KM curves produced a lower ICER than the base-case scenario (SG$129,416 vs. SG$137,648 per QALY). Conversely, applying an exponential parametric fit to the model produced a higher ICER of SG$143,658 per QALY. The pricing analyses showed that reducing the cost of afatinib by 10% to 50% lowered the ICER to SG$128,348 - SG$91,147 per QALY. None of the scenario analyses brought the ICER into an acceptable range of cost-effectiveness in Singapore’s context.

**Table 7:** Summary of cost and benefit in the additional scenario analysis

	Cost(SG$)	QALYs	LYs	PFLYs	ICER (SG$/QALY)
Independent model with parametric fitting^a^					
Weilbull					
PemCis	76,679	1.54	2.38	0.66	
Afatinib	92,486	1.66	2.55	1.10	129,416
Exponential					
PemCis	73,775	1.53	2.37	0.71	
Afatinib	92,507	1.66	2.55	1.18	143,658
Log-normal					
PemCis	76,851	1.57	2.43	0.70	
Afatinib	94,966	1.72	2.61	1.32	126,202
Log-logistic					
PemCis	76,046	1.57	2.43	0.73	
Afatinib	94,764	1.71	2.60	1.34	133,627
Pricing scenario^b^					
10% reduction					
PemCis	75,682	1.58	2.45	0.68	
Afatinib	89,880	1.69	2.59	1.18	128,348
20% reduction					
PemCis	72,632	1.58	2.45	0.68	
Afatinib	85,802	1.69	2.59	1.18	119,048
30% reduction					
PemCis	69,583	1.58	2.45	0.68	
Afatinib	81,724	1.69	2.59	1.18	109,747
40% reduction					
PemCis	66,534	1.58	2.45	0.68	
Afatinib	77,646	1.69	2.59	1.18	100,447
50% reduction					
PemCis	63,485	1.58	2.45	0.68	
Afatinib	73,568	1.69	2.59	1.18	91,147

*ICER* incremental cost-effectiveness ratio, *QALY* quality-adjusted life year, *LY* life year, *PFLY* progression-free life year, *PemCis* pemetrexed-cisplatin

^a^Progression-free and overall survival curves of both intervention arms extrapolated from Kaplan-Meier data from trial, with parametric curve fitting from time = 0 to tail-end

^b^Pricing scenario with various discounting on the cost of afatinib. Afatinib was modelled in the first-line for the afatinib arm, and post-progression (second-line) for the PemCis arm, therefore the total cost of both arms reduced as a result of the reduction in selling price of afatinib

## Discussion

In Singapore, there is increasing use of afatinib and other TKIs for the treatment of NSCLC, due to their improved progression-free survival outcomes, favourable AE profiles and the convenience they offer patients through oral administration compared to chemotherapy. However, TKIs are substantially more costly than platinum doublet chemotherapy, making them largely unaffordable for most patients. To our best knowledge, this is the first study conducted to address the cost-effectiveness of afatinib as first-line therapy for patients with EGFR mutation-positive NSCLC in Singapore. It complements a previous cost-effectiveness analysis conducted in Singapore that suggested EGFR mutation testing coupled with gefitinib was the dominant treatment strategy, compared with no mutation testing plus chemotherapy [22].

Clinical trials informing this study consistently showed no statistically significant improvement in OS for afatinib compared with platinum-based chemotherapy for EGFR mutation-positive NSCLC, although significant improvements in PFS were demonstrated. These results are also consistent with studies of other TKIs (erlotinib and gefitinib) compared with platinum doublet chemotherapy [5, 6, 8–12, 23–28]. Although OS remains the gold standard metric of benefit for clinical trials involving therapeutic oncology agents, successive lines of treatment, patient crossover and increased post-progression survival may dilute treatment effects. While surrogate endpoints such as PFS potentially offer more feasible options to measure clinical benefit, allowing for shorter trial duration and smaller patient cohorts, the correlation between PFS and OS requires validation given it is context dependent and contingent on disease, stage, patient population and therapy [29].

Investigator-reviewed rather than independent-reviewed PFS curve was used in our base-case analysis to mimic patient assessments in real-world clinical practice. We acknowledge that there are limitations with both approaches, therefore selection of appropriate estimates requires careful consideration. Although independent-review of PFS may lessen some potential investigator biases, it can introduce informative censoring [30, 31]. On the other hand, meta-analysis shows that investigator-review can provide reliable estimates with little evidence of systematic evaluation bias [32]. Both investigator-review (median PFS of 11.1 months for afatinib, 6.7 months for PemCis) and independent-review (median PFS of 11.1 months for afatinib, 6.9 months for PemCis) curves yielded similar PFS results in LUX-Lung 3 [6]. For our CEA, any uncertainty of PFS was further addressed by probabilistic sensitivity analysis and scenario analyses with different survival curve extrapolation approaches, therefore the choice of curve was not considered to have a material impact on our results.

In cost-effectiveness modelling, when applying a parametric survival curve to the tail-end of empirical KM data, jump-off bias is a common phenomenon where the observed KM curve and the extrapolated or forecasted fitted parametric curve do not join smoothly. This is most evident when running PSA where the forecasted OS value randomly sampled for a particular month may be unrealistically higher than the previous month, if the reduction factor was not applied. In our base-case analysis, a jump-off bias adjustment was crucial because the PFS and OS survival curves from the trial were relatively short (22 & 49 months respectively). To tackle this issue, reduction factors were applied to both OS and PFS curves to combine the observed data and the extrapolated tail-end data to prevent any jump-off bias and in turn generate a ‘smooth’ curve. For instance, OS in each forecasted month was calculated as the product of the OS estimated by the Weilbull model, and the ratio of the last observed OS value in the curve and the OS estimated by the Weilbull model for the last observed OS value.

It is worth noting that the cost of the PD health state for both intervention arms was higher than for the PF health state, largely driven by the cost and assumed substantial duration of third-line treatment with BSC. Patients receiving first-line afatinib crossed over to second-line PemCis on progression which was associated with a shorter PFS period of 5.4 months, and resulted in a long duration (16 months) of BSC on further disease progression. Patients treated with first-line PemCis received BSC third-line for 11.5 months in the model.

Our base-case analysis reveals that afatinib is not a cost-effective treatment option at its current price. A key contributor to the high ICER value was the cost of afatinib relative to PemCis. This was reflected in the incremental difference in first-line drug cost of SG$34,783, which was driven by the longer PFS duration associated with afatinib, thus accounting for more daily doses of afatinib until progression, whereas PemCis costs were capped at 6 cycles. Similarly, as patients receiving first-line PemCis were assumed to cross-over to afatinib second-line on progression, the cost of the PD health state in the PemCis arm was thus higher than the afatinib arm. Although different pricing scenarios were tested, even reducing the cost of afatinib by 50% only lowered the ICER to SG$91,147 per QALY gained, which is unlikely to be considered cost-effective in the Singapore setting.

Our results are comparable with published ICERs for TKIs in overseas settings. An analysis of first-line treatment of patients with EGFR mutation-positive NSCLC in the UK reported an ICER for gefitinib compared with PemCis ranging from £23,615 (maximum of 6 cycles) to £64,481 per QALY gained (a maximum of 5 cycles). The analysis used a Markov economic model and a similar 5-year time horizon [33]. Ting et al. [34] assessed the cost-effectiveness of erlotinib, afatinib and PemCis for first-line treatment of advanced epithelial EGFR mutation-positive NSCLC in the US. The authors also suggested erlotinib was more effective and more costly compared with PemCis, with an ICER of US$40,106 per QALY gained. However, contrary to our results, afatinib was found to be cost saving in the US, largely due to the fact that it had the lowest cost price (followed by PemCis and then erlotinib) while in Singapore, PemCis is the least expensive treatment option, followed by erlotinib, then afatinib based on current cost prices.

One-way sensitivity analysis was performed to assess the key drivers of the model by varying the input parameters. The key driver of ICER was the utility values used, which were extracted from published literature due to the absence of local data. There is little published data on the related preferences of patients in different health states during NSCLC disease progression, and this is a key limitation of our study. For the purposes of our analysis, we adapted utility values from a multi-country, quality-of-life survey of patients with advanced NSCLC, and applied disutility values associated with AEs which were sourced from another study conducted in the UK general public. Combining the utility values from two studies consisting of different populations is not ideal and may affect the validity of the results.

Our model had a lifetime horizon of 5 years in the base case, which was similar to the published CEA conducted in the UK [31]. This time period was considered clinically plausible given Singapore’s cancer registry [1] showed that the 5-year age-standardised observed survival rate for stage IV lung cancer is low (3.14% for men and 4.82% for women). Sensitivity analysis confirmed that the model was sensitive to the time horizon. Prolonging the time horizon to 10 years reduced the ICER considerably because the longer time horizon allowed for all QALYs to be fully captured. The increase in QALY was driven by the increase in LY while the PFLY remained constant.

There are several limitations of our economic model that may affect its robustness. Firstly, both utilities studies which informed inputs in the model were conducted in western countries (9% of the population were Asian in the study by Chouaid et al. [21], whereas the ethnicity of the study population in Nafees et al. [22] was not reported); thus, utility values may not be wholly generalisable to Asian patients in Singapore. Secondly, the model closely mimicked the trial design – i.e. after receiving first-line afatinib or PemCis, patients were allowed to switch to subsequent therapy upon disease progression. Hence, the model did not allow for the use of maintenance treatments such as erlotinib or pemetrexed which may not be true to local clinical practice.

Thirdly, the PFS data for second-line afatinib and PemCis were derived from published trials which were thought to best fit the treatment sequence employed in the model. No published data that was identical to the treatment sequencing in the model was available. In addition, local clinical guidelines recommend pemetrexed-carboplatin and pemetrexed-cisplatin chemotherapy for the first-line treatment of non-squamous advanced NSCLC in view of its better outcome than platinum doublet chemotherapy without pemetrexed. However, our analysis did not include pemetrexed-carboplatin as a comparator to afatinib, in the absence of trial data.

A further limitation of our study was the lack of cost data for AEs treated in an outpatient setting. Excluding the cost of neutropenia for example may overestimate the ICER given the rate of neutropenia was reported to be higher in the PemCis treatment arm of LUX-Lung 3. In Singapore, it is likely that patients who experience neutropenia may not require hospitalisation and could be managed in an outpatient setting with granulocyte colony growth factors (G-CSF). Similarly, LUX-Lung 3 reported higher rates of diarrhoea and rash in the afatinib treatment arm. The cost of outpatient therapies for these AEs, such anti-diarrhoea tablet, hydration solution, moisturiser and antihistamine were not included in the model. For our CEA, only the costs of AEs that necessitated inpatient hospitalisation were included. The cost of G-CSF and other over-the-counter medications were considered negligible because they had a marginal overall cost impact compared to hospital admission. In addition, the cost of G-CSF is halved since the entry of biosimilar in 2014 and its cost is expected to continue to decline given multiple biosimilars are now available.

Lastly, our analysis did not account for vial sharing of pemetrexed. Hence this would tend to underestimate the ICER given that vial sharing is common practice in Singapore as most patients are treated in one of the two large specialised cancer centres within the public healthcare institutions.

## Conclusions

TKIs remain a very costly first-line treatment option for advanced EGFR mutation-positive NSCLC as currently none of the TKIs have been shown to improve overall survival. Our analysis showed that afatinib is not a cost-effective first-line treatment in Singapore and does not represent good value for limited health care dollars compared with pemetrexed-cisplatin. The findings from our study will be useful to inform local healthcare decision-making and resource allocations for NSCLC treatments, together with other considerations such as clinical effectiveness, safety and affordability of TKIs.

## References

[1] Singapore Cancer Registry Annual Registry Report 2015. Released 19 June 2017. https://www.nrdo.gov.sg/docs/librariesprovider3/Publications-Cancer/cancer-registry-annual-report-2015_web.pdf?sfvrsn=6. Accessed 20 July 2017.

[2] Besse B, Adjei A, Baas P, Meldgaard P, Nicolson M, Paz-Ares L, Reck M, Smit EF, Syrigos K, Stahel R (2014). 2nd ESMO consensus conference on lung Cancer: non-small-cell lung cancer first-line/second and further lines of treatment in advanced disease. Ann Oncol.

[3] Reck M, Popat S, Reinmuth N, De Ruysscher D, Kerr KM, Peters S, Group EGW (2014). Metastatic non-small-cell lung cancer (NSCLC): ESMO clinical practice guidelines for diagnosis, treatment and follow-up. Ann Oncol.

[4] Shi Y, Au JS, Thongprasert S, Srinivasan S, Tsai CM, Khoa MT, Heeroma K, Itoh Y, Cornelio G, Yang PC (2014). A prospective, molecular epidemiology study of EGFR mutations in Asian patients with advanced non-small-cell lung cancer of adenocarcinoma histology (PIONEER). J Thorac Oncol.

[5] Mitsudomi T, Morita S, Yatabe Y, Negoro S, Okamoto I, Tsurutani J, Seto T, Satouchi M, Tada H, Hirashima T (2010). Gefitinib versus cisplatin plus docetaxel in patients with non-small-cell lung cancer harbouring mutations of the epidermal growth factor receptor (WJTOG3405): an open label, randomised phase 3 trial. Lancet Oncol.

[6] Sequist LV, Yang JC, Yamamoto N, O'Byrne K, Hirsh V, Mok T, Geater SL, Orlov S, Tsai CM, Boyer M (2013). Phase III study of afatinib or cisplatin plus pemetrexed in patients with metastatic lung adenocarcinoma with EGFR mutations. J Clin Oncol.

[7] Singapore Lung Cancer Workgroup. Singapore Cancer Network (SCAN) Guidelines for the Use of Systemic Therapy in Advanced Non-Small Cell Lung Cancer. Ann Acad Med Singap. 2015;44:449–62. 26763063

[8] Wu YL, Zhou C, Liam CK, Wu G, Liu X, Zhong Z, Lu S, Cheng Y, Han B, Chen L (2015). First-line erlotinib versus gemcitabine/cisplatin in patients with advanced EGFR mutation-positive non-small-cell lung cancer: analyses from the phase III, randomized, open-label, ENSURE study. Ann Oncol.

[9] Rosell R, Carcereny E, Gervais R, Vergnenegre A, Massuti B, Felip E, Palmero R, Garcia-Gomez R, Pallares C, Sanchez JM (2012). Erlotinib versus standard chemotherapy as first-line treatment for European patients with advanced EGFR mutation-positive non-small-cell lung cancer (EURTAC): a multicentre, open-label, randomised phase 3 trial. Lancet Oncol.

[10] Fukuoka M, Wu YL, Thongprasert S, Sunpaweravong P, Leong SS, Sriuranpong V, Chao TY, Nakagawa K, Chu DT, Saijo N (2011). Biomarker analyses and final overall survival results from a phase III, randomized, open-label, first-line study of gefitinib versus carboplatin/paclitaxel in clinically selected patients with advanced non-small-cell lung cancer in Asia (IPASS). J Clin Oncol.

[11] Park K, Tan E-H, O'Byrne K, Zhang L, Boyer M, Mok T, Hirsh V, Yang JC-H, Lee KH, Lu S (2016). Afatinib versus gefitinib as first-line treatment of patients with EGFR mutation-positive non-small-cell lung cancer (LUX-lung 7): a phase 2B, open-label, randomised controlled trial. Lancet Oncol.

[12] Yang JC-H, Wu Y-L, Schuler M, Sebastian M, Popat S, Yamamoto N, Zhou C, Hu C-P, O'Byrne K, Feng J (2015). Afatinib versus cisplatin-based chemotherapy for EGFR mutation-positive lung adenocarcinoma (LUX-lung 3 and LUX-lung 6): analysis of overall survival data from two randomised, phase 3 trials. Lancet Oncol.

[13] Soria J-C, Wu Y-L, Nakagawa K, Kim S-W, Yang J-J, Ahn M-J, Wang J, Yang JC-H, Lu Y, Atagi S (2015). Gefitinib plus chemotherapy versus placebo plus chemotherapy in EGFR-mutation-positive non-small-cell lung cancer after progression on first-line gefitinib (IMPRESS): a phase 3 randomised trial. Lancet Oncol.

[14] Kim ST, Uhm JE, Lee J, Sun JM, Sohn I, Kim SW, Jung SH, Park YH, Ahn JS, Park K, Ahn MJ (2012). Randomized phase II study of gefitinib versus erlotinib in patients with advanced non-small cell lung cancer who failed previous chemotherapy. Lung Cancer.

[15] Rohatgi A. WebPlotDigitizer Version 3.9. http://arohatgi.info/WebPlotDigitizer. Accessed 5 Dec 2016.

[16] Hoyle MW, Henley W (2011). Improved curve fits to summary survival data: application to economic evaluation of health technologies. BMC Med Res Methodol.

[17] Renshaw AE, Haberman S (2003). On the forecasting of mortality reduction factors. Insur Math Econ.

[18] Ministry of Health. Total Hospital bills - by condition/procedure. https://www.moh.gov.sg/content/moh_web/home/costs_and_financing/hospital-charges/Total-Hospital-Bills-By-condition-procedure.html. Accessed 15 Nov 2016.

[19] Ministry of Health. Average hospital inpatient bill size tables. https://www.moh.gov.sg/content/moh_web/home/costs_and_financing/average_hospitalinpatientbillsizetables.html. Accessed 15 Nov 2016.

[20] Chouaid C, Agulnik J, Goker E, Herder GJ, Lester JF, Vansteenkiste J, Finnern HW, Lungershausen J, Eriksson J, Kim K, Mitchell PL (2013). Health-related quality of life and utility in patients with advanced non-small-cell lung cancer: a prospective cross-sectional patient survey in a real-world setting. J Thorac Oncol.

[21] Nafees B, Stafford M, Gavriel S, Bhalla S, Watkins J (2008). Health state utilities for non small cell lung cancer. Health Qual Life Outcomes.

[22] de Lima Lopes G, Segel JE, Tan DS, Do YK, Mok T, Finkelstein EA (2012). Cost-effectiveness of epidermal growth factor receptor mutation testing and first-line treatment with gefitinib for patients with advanced adenocarcinoma of the lung. Cancer.

[23] Zhou C, Wu YL, Chen G, Feng J, Liu XQ, Wang C, Zhang S, Wang J, Zhou S, Ren S (2015). Final overall survival results from a randomised, phase III study of erlotinib versus chemotherapy as first-line treatment of EGFR mutation-positive advanced non-small-cell lung cancer (OPTIMAL, CTONG-0802). Ann Oncol.

[24] Zhou C, Wu Y-L, Chen G, Feng J, Liu X-Q, Wang C, Zhang S, Wang J, Zhou S, Ren S (2011). Erlotinib versus chemotherapy as first-line treatment for patients with advanced EGFR mutation-positive non-small-cell lung cancer (OPTIMAL, CTONG-0802): a multicentre, open-label, randomised, phase 3 study. Lancet Oncol.

[25] Han JY, Park K, Kim SW, Lee DH, Kim HY, Kim HT, Ahn MJ, Yun T, Ahn JS, Suh C (2012). First-SIGNAL: first-line single-agent iressa versus gemcitabine and cisplatin trial in never-smokers with adenocarcinoma of the lung. J Clin Oncol.

[26] Gridelli C, Ciardiello F, Gallo C, Feld R, Butts C, Gebbia V, Maione P, Morgillo F, Genestreti G, Favaretto A (2012). First-line erlotinib followed by second-line cisplatin-gemcitabine chemotherapy in advanced non-small-cell lung cancer: the TORCH randomized trial. J Clin Oncol.

[27] Inoue A, Kobayashi K, Maemondo M, Sugawara S, Oizumi S, Isobe H, Gemma A, Harada M, Yoshizawa H, Kinoshita I (2013). Updated overall survival results from a randomized phase III trial comparing gefitinib with carboplatin-paclitaxel for chemo-naive non-small cell lung cancer with sensitive EGFR gene mutations (NEJ002). Ann Oncol.

[28] Yoshioka H, Tetsuya M, Morita S (2014). Final overall survival results of WJTOG 3405, a randomized phase 3 trial comparing gefitinib (G) with cisplatin plus docetaxel (CD) as the firstline treatment for patients with nonsmall cell lung cancer (NSCLC) harboring mutations of the epidermal growth factor receptor (EGFR). J Clin Oncol.

[29] Wilson MK, Karakasis K, Oza AM (2015). Outcomes and endpoints in trials of cancer treatment: the past, present, and future. Lancet Oncol.

[30] Korn RL, Crowley JJ (2013). Overview: progression-free survival as an endpoint in clinical trials with solid tumors. Clin Cancer Res.

[31] Dodd LE, Korn EL, Freidlin B, Jaffe CC, Rubinstein LV, Dancey J, Mooney MM (2008). Blinded independent central review of progression-free survival in phase III clinical trials: important design element or unnecessary expense?. J Clin Oncol.

[32] Amit O, Mannino F, Stone AM, Bushnell W, Denne J, Helterbrand J, Burger HU (2011). Blinded independent central review of progression in cancer clinical trials: results from a meta-analysis. Eur J Cancer.

[33] Brown T, Boland A, Bagust A, Oyee J, Hockenhull J, Dundar Y, Dickson R, Ramani VS, Proudlove C (2010). Gefitinib for the first-line treatment of locally advanced or metastatic non-small cell lung cancer. Health Technol Assess.

[34] Ting J, Tien Ho P, Xiang P, Sugay A, Abdel-Sattar M, Wilson L (2015). Cost-effectiveness and value of information of Erlotinib, Afatinib, and cisplatin-Pemetrexed for first-line treatment of advanced EGFR mutation-positive non-small-cell lung Cancer in the United States. Value Health.

